# Ketamine and Sodium Selenite Promote Transient Receptor Potential Vanilloid 1-Associated Calcium Signaling and Apoptosis in SH-SY5Y Neuroblastoma Cells

**DOI:** 10.3390/cimb48090870

**Published:** 2026-08-27

**Authors:** Ebru Aladag, Ishak Suat Ovey

**Affiliations:** 1 Department of Anesthesiology and Reanimation, Faculty of Medicine, Alanya Alaaddin Keykubat University, Alanya 07425, Turkey; 2 Department of Physiology, Faculty of Medicine, Alanya Alaaddin Keykubat University, Alanya 07425, Turkey; suat.ovey@alanya.edu.tr

**Keywords:** apoptosis, calcium signaling, ketamine, neuroblastoma, oxidative stress, sodium selenite, TRPV1 channel, SH-SY5Y

## Abstract

Ketamine has pharmacological actions beyond N-methyl-D-aspartate receptor antagonism, whereas sodium selenite modulates redox-dependent signaling. Transient receptor potential vanilloid 1 (TRPV1) channels regulate Ca^2+^ influx and may contribute to oxidative stress-associated apoptosis. We investigated whether ketamine and sodium selenite, alone and combined, promote apoptosis through TRPV1-associated mechanisms in SH-SY5Y neuroblastoma cells. Cells were assigned to control, ketamine+sodium selenite (Ket+Sel), Ket+Sel+capsazepine (CPZ), ketamine, ketamine+CPZ, sodium selenite, and sodium selenite+CPZ groups. Ketamine (10 µM) and/or sodium selenite (500 nM) were applied for 48 h; CPZ (0.1 mM, 30 min) was used for pharmacological antagonism, and capsaicin (0.1 mM) for stimulation. Intracellular Ca^2+^ responses were assessed using Fura-2-AM, whereas apoptosis, reactive oxygen species production, mitochondrial depolarization, and caspase-3/9 activities were measured spectrophotometrically or fluorometrically. Under capsaicin-stimulated assay conditions, ketamine and sodium selenite increased all endpoints relative to control, and the combined treatment produced the greatest responses for most endpoints. CPZ attenuated Ca^2+^ responses and several downstream oxidative and apoptotic markers. These findings indicate that ketamine and sodium selenite, particularly in combination, enhance apoptosis through a CPZ-sensitive pathway compatible with TRPV1-associated Ca^2+^-ROS-mitochondrial signaling, while not establishing exclusive TRPV1 mediation.

## 1. Introduction

Neuroblastoma is an embryonal tumor derived from neural crest cells and is one of the most common extracranial solid tumors in childhood. The disease is characterized by marked biological, histopathological, and clinical heterogeneity, with disease behavior ranging from spontaneous regression to aggressive metastatic progression [1,2]. This heterogeneity is reflected in differences in histologic category and degree of tumor differentiation, together with molecular prognostic features such as MYCN status, 11q aberration, and DNA ploidy, which underpin contemporary risk stratification and are associated with prognosis [1,2]. Despite multimodal treatment strategies that include surgery, chemotherapy, high-dose chemotherapy with autologous stem-cell rescue, and radiotherapy, outcomes remain unsatisfactory in many patients with high-risk disease [1,2,3]. Therefore, experimental approaches that clarify mechanisms of cancer cell death remain important for identifying potential adjunctive targets.

Ketamine is a well-characterized anesthetic and analgesic drug whose pharmacology extends beyond non-competitive antagonism of the N-methyl-D-aspartate receptor [4]. Ketamine interacts with multiple receptor and ion-channel systems, including opioid, cholinergic, monoaminergic, sodium, and potassium channel pathways [4,5]. Experimental evidence indicates that ketamine can influence cell survival, mitochondrial signaling, and apoptosis in neuronal and cancer cell models [6,7,8,9]. In SH-SY5Y neuroblastoma cells, ketamine has been investigated for its effects on cell viability, primary DNA damage, and oxidative stress parameters, suggesting that its cellular actions may be relevant to neuroblastoma biology [10].

Dysregulated intracellular Ca^2+^ homeostasis can affect cancer-cell proliferation, apoptosis, metabolism, migration, and invasion [11]. Cytosolic Ca^2+^ signaling is regulated by both Ca^2+^ entry across the plasma membrane and Ca^2+^ mobilization from intracellular stores. An important mechanism underlying intracellular Ca^2+^ mobilization is phosphoinositide-specific phospholipase C (PI-PLC) signaling. PI-PLC-mediated hydrolysis of phosphatidylinositol 4,5-bisphosphate generates the second messengers inositol 1,4,5-trisphosphate (IP_3_) and diacylglycerol, with IP_3_ mobilizing Ca^2+^ from intracellular stores [12]. PI-PLC isoforms also participate in diverse signaling processes in the nervous system, and dysregulation of PI-PLC signaling has been associated with several neurological disorders [12]. In glioblastoma, PLCβ1 expression has been reported to inversely correlate with glioma grade, while experimental PLCβ1 silencing increased migration and invasion and altered proliferative and survival-associated signaling pathways [13]. Within this broader framework of intracellular Ca^2+^ regulation, transient receptor potential (TRP) channels contribute to Ca^2+^ signaling and have been implicated in multiple stages of cancer progression [11]. Among these channels, TRPV1 is a Ca^2+^-permeable channel, and TRPV1-associated Ca^2+^ signaling has been linked to oxidative stress and apoptosis in a neuronal model [14] and to Ca^2+^ overload, ROS generation, mitochondrial dysfunction, and apoptotic cell death in cancer cells [15].

Selenium is an essential trace element involved in redox regulation and antioxidant defense [16]. Depending on concentration and cellular context, selenium compounds may exert either antioxidant and cytoprotective effects or pro-oxidant and pro-apoptotic effects in different cellular models [17,18]. Previous studies have shown that selenium can modulate Ca^2+^ entry and signaling, oxidative stress, and apoptosis through TRPV1-associated pathways in different cellular models [19,20]. These findings provide a rationale for examining sodium selenite as a redox-active modulator of TRPV1-related apoptosis in neuroblastoma cells.

**Figure 1:**
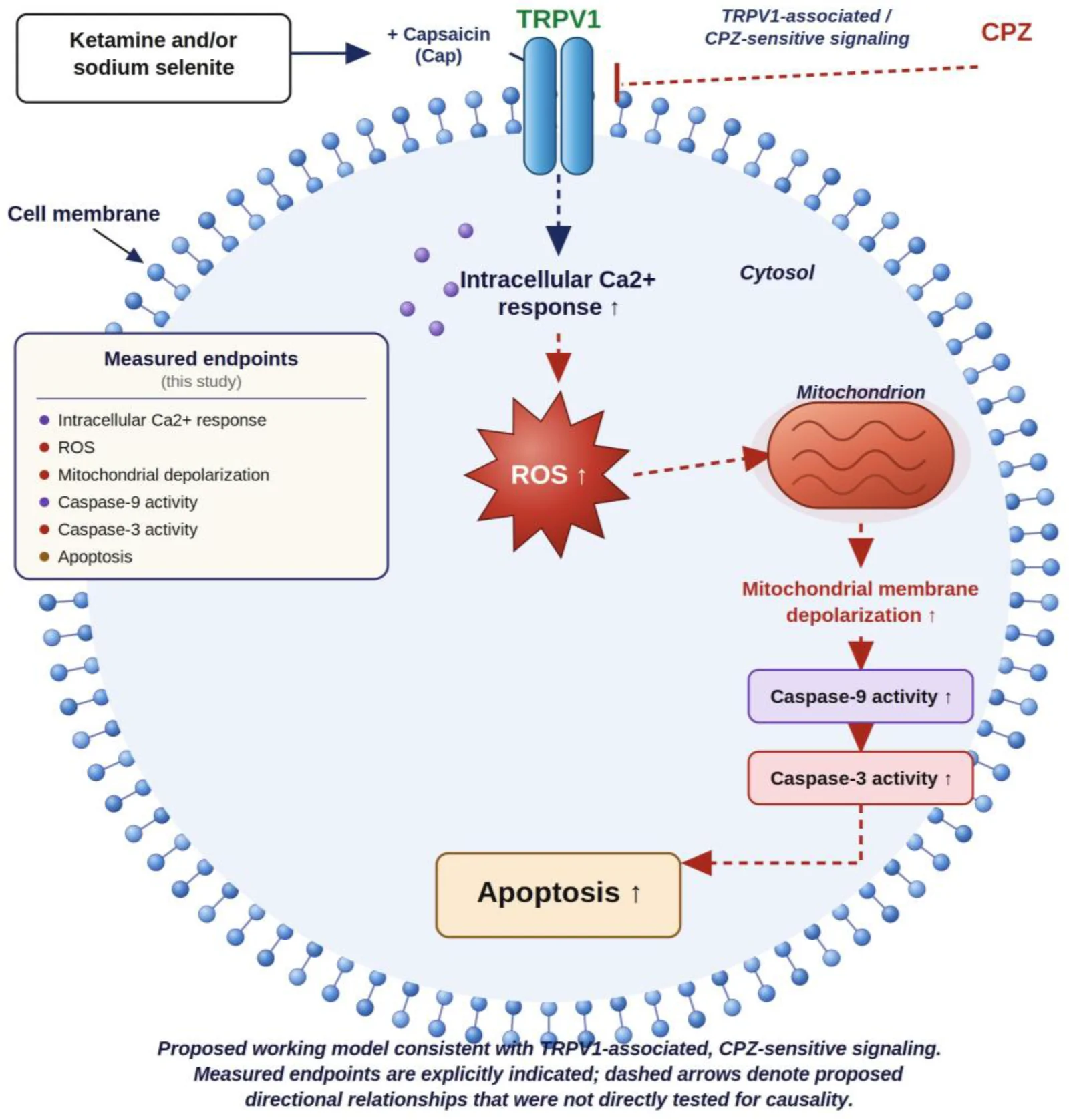
Proposed working model of the responses observed after ketamine and/or sodium selenite exposure in capsaicin-stimulated SH-SY5Y neuroblastoma cells, consistent with TRPV1-associated, CPZ-sensitive signaling. Cells were exposed to ketamine and/or sodium selenite, with or without capsazepine (CPZ) pretreatment, followed by capsaicin (Cap) stimulation. Dashed arrows indicate proposed directional relationships among TRPV1-associated signaling, intracellular Ca^2+^ responses, ROS production, mitochondrial membrane depolarization, caspase activity, and apoptosis; these step-wise causal relationships were not directly tested. Attenuation of several responses by CPZ is compatible with a CPZ-sensitive, TRPV1-associated signaling component but does not establish exclusive TRPV1 mediation. Experimentally assessed endpoints included intracellular Ca^2+^ response, ROS production, mitochondrial membrane depolarization, caspase-9 activity, caspase-3 activity, and apoptosis. Upward arrows indicate increased responses; colors are used only for visual differentiation. CPZ, capsazepine; ROS, reactive oxygen species; TRPV1, transient receptor potential vanilloid 1.

Although ketamine and sodium selenite have independently been associated with alterations in intracellular Ca^2+^ signaling, redox homeostasis, mitochondrial function, and apoptosis [4,5,7,8,17,18,19,20], there is a clear biological rationale for examining their combined effects. Ketamine can modulate ion-channel activity and mitochondrial signaling [4,5,7,8], whereas sodium selenite can alter cellular redox status and Ca^2+^-dependent signaling pathways [17,18,19,20]. Because disturbances in intracellular Ca^2+^ homeostasis and redox balance can converge on mitochondrial dysfunction and caspase-dependent apoptotic signaling [11,15,17,20], we hypothesized that concurrent exposure to ketamine and sodium selenite might enhance apoptotic responses through partially overlapping Ca^2+^-redox–mitochondrial mechanisms. This potential mechanistic convergence prompted us to evaluate the combined treatment in SH-SY5Y neuroblastoma cells. The present study evaluated the effects of ketamine and sodium selenite, alone and in combination, on intracellular Ca^2+^ responses, ROS production, mitochondrial membrane depolarization, apoptosis, and caspase-3 and caspase-9 activities, and examined whether TRPV1-associated signaling might contribute to these responses using pharmacological modulation. The proposed working model is illustrated in Figure 1.

## 2. Materials and Methods

### 2.1. Reagents and Stains

Dulbecco’s Modified Eagle Medium (DMEM), Ham’s F12 medium, trypsin-EDTA, fetal bovine serum (FBS), penicillin-streptomycin, dimethyl sulfoxide (DMSO), capsaicin, and dihydrorhodamine-123 (DHR-123) were obtained from Sigma-Aldrich (St. Louis, MO, USA). Capsazepine was obtained from Cayman Chemical (Ann Arbor, MI, USA). Sodium selenite was obtained from Carl Roth (Karlsruhe-Mühlburg, Germany). Ketamine was obtained from Pfizer (New York, NY, USA). Fura-2 acetoxymethyl ester (Fura-2-AM) was purchased from Calbiochem (Darmstadt, Germany), and Pluronic F-127 was obtained from BioVision (San Francisco, CA, USA). The APOPercentage apoptosis assay kit and release buffer were obtained from Biocolor Ltd. (Belfast, Northern Ireland). Probenecid and JC-1 were purchased from Santa Cruz Biotechnology (Dallas, TX, USA). Caspase-3 (Ac-DEVD-AMC) and caspase-9 (Ac-LEHD-AMC) fluorogenic substrates were supplied by Enzo Life Sciences (Lausanne, Switzerland). Capsaicin and capsazepine stock solutions were prepared in DMSO. Sodium selenite was dissolved in distilled water.

### 2.2. Cell Culture

Human SH-SY5Y neuroblastoma cells were obtained from the American Type Culture Collection (ATCC; Manassas, VA, USA). Cells were cultured in a 1:1 mixture of Ham’s F12 medium and DMEM supplemented with 10% FBS and 1% penicillin-streptomycin. SH-SY5Y cells at passages 10, 11, and 13 (P10, P11, and P13) were maintained in sterile T25 flasks at 37 °C in a humidified incubator with 5% CO_2_. Cells were seeded at approximately 4 × 10^5^ cells per T25 flask. Cells were monitored daily for contamination and used for experiments at 75–85% confluence.

Following treatment, cells were washed with phosphate-buffered saline, detached using 0.25% trypsin-EDTA, collected into 15 mL conical tubes, and centrifuged at 100× *g* for 5 min. Supernatants were removed, and cell pellets were resuspended in fresh medium for subsequent analyses.

### 2.3. Experimental Groups

SH-SY5Y cells were divided into seven experimental groups.

Group 1 (Control): The cells were not incubated with ketamine, sodium selenite, or capsazepine (CPZ) but were maintained in the same cell culture medium and under the same conditions.

Group 2 (Ket+Sel): Cells in the group were incubated with 10 µM ketamine and 500 nM sodium selenite for 48 h [21,22].

Group 3 (Ket+Sel+CPZ): Cells in the group were incubated with 10 µM ketamine and 500 nM sodium selenite for 48 h [21,22] and then incubated with capsazepine (TRPV1 channel antagonist, CPZ, 0.1 mM, 30 min).

Group 4 (Ket): Cells in the group were incubated with 10 µM ketamine for 48 h [21].

Group 5 (Ket+CPZ): Cells in the group were incubated with 10 µM ketamine for 48 h [21] and then incubated with capsazepine (TRPV1 channel antagonist, CPZ, 0.1 mM, 30 min).

Group 6 (Sel): Cells in the group were incubated with 500 nM sodium selenite for 48 h [22].

Group 7 (Sel+CPZ): Cells in the group were incubated with 500 nM sodium selenite for 48 h [22] and then incubated with capsazepine (TRPV1 channel antagonist, CPZ, 0.1 mM, 30 min).

Ketamine (Ket) was applied at 10 µM and sodium selenite (Sel) at 500 nM for 48 h. The 48 h ketamine exposure was selected with reference to previous in vitro work in SH-SY5Y cells [21]. The sodium selenite treatment condition (500 nM, 48 h) was selected based on previous in vitro work [22]. Capsazepine (CPZ; 0.1 mM, 30 min) was used as a TRPV1 antagonist. For intracellular Ca^2+^ measurements, capsaicin (Cap; 0.1 mM) was applied at the 20th acquisition cycle, and changes in intracellular Ca^2+^ levels were monitored using the Fura-2-AM ratiometric fluorescence method. For apoptosis, ROS, mitochondrial depolarization, and caspase assays, cells were stimulated with Cap (0.1 mM, 10 min) before measurements. Capsaicin and capsazepine were used as pharmacological modulators of TRPV1 based on a previously published experimental approach [20]. A separate vehicle-control group was not included.

### 2.4. Measurement of Intracellular Free Calcium Concentration

Intracellular free Ca^2+^ concentration ([Ca^2+^]_i_) was measured using the UV-excitable ratiometric calcium indicator Fura-2-AM, with modifications based on previously published methods [17,23]. After treatment, cells were incubated with 5 µM Fura-2-AM and 0.05% Pluronic F-127 in HEPES-buffered saline at 37 °C in the dark. After washing and de-esterification, cells were seeded into black, clear-bottom 96-well plates. Fluorescence emission at 510 nm was recorded for 50 acquisition cycles at alternating excitation wavelengths of 340 and 380 nm using a Synergy H1 microplate reader (BioTek, Winooski, VT, USA). Cap (0.1 mM) was applied using the automated injector at the 20th cycle. Intracellular Ca^2+^ responses were expressed as the F340/F380 ratio or fold change relative to control values.

### 2.5. Assessment of Apoptosis and Intracellular ROS Production

Apoptosis was evaluated using the APOPercentage assay (Biocolor Ltd.) according to the manufacturer’s instructions and previously published methods [24,25]. The assay stains apoptotic cells based on membrane asymmetry changes and phosphatidylserine exposure. Absorbance was measured at 550 nm using a Synergy H1 microplate reader.

For the determination of intracellular reactive oxygen species (ROS) production, three separate 1 mL cell suspensions, each containing 10^6^ isolated cells in 1× PBS, were prepared in three Eppendorf tubes [24,25]. From these suspensions, 150 µL was transferred into each of two Eppendorf tubes, while 200 µL was transferred into the third tube. Subsequently, 850 µL of 1× PBS was added to each of the first two tubes, and 800 µL of 1× PBS was added to the third tube, resulting in a final volume of 1 mL in each Eppendorf tube. The samples were then gently homogenized by 4–5 cycles of pipetting. Subsequently, dihydrorhodamine 123 (DHR-123; Sigma-Aldrich, MO, USA) was added to the cell suspensions at a final concentration of 2 µM, followed by another 4–5 gentle pipetting cycles to ensure adequate mixing. The Eppendorf tubes containing the cell suspension and DHR-123 were then incubated for 30 min at 37 °C in a shaking water bath. Following incubation, the samples were centrifuged at 100× *g* for 5 min. The supernatants were discarded, and 300 µL of 1× PBS was added to two tubes, while 400 µL of 1× PBS was added to the third tube. The cells were gently resuspended by pipetting. Subsequently, 100 µL of each sample (*n* = 10), corresponding to approximately 5 × 10^4^ cells per well, was transferred into the wells of a microplate. Fluorescence intensity was measured using a Synergy™ H1 microplate reader at excitation and emission wavelengths of 488 and 543 nm, respectively. The data were expressed as fold changes relative to the control or pretreatment values.

### 2.6. Caspase-3 and Caspase-9 Activity Assays

Caspase-3 and caspase-9 activities were measured using Ac-DEVD-AMC and Ac-LEHD-AMC substrates, respectively, according to previously described methods [24,25]. Cell lysates were incubated with the appropriate substrate solution at 37 °C. Fluorescence was measured at excitation and emission wavelengths of 360 and 460 nm, respectively, using a Synergy H1 microplate reader. Total protein concentrations were determined using the Bradford protein assay. Caspase-3 and caspase-9 activities were normalized to total protein content and expressed as fluorescence units per milligram of protein. The normalized values were expressed as fold change relative to control values.

### 2.7. Mitochondrial Membrane Potential Analysis

Mitochondrial membrane potential was assessed using JC-1 dye [24,25]. Following treatment, SH-SY5Y cells were incubated with JC-1 (1 µM) for 45 min at 37 °C. Green fluorescence was measured at 485/535 nm and red fluorescence at 540/590 nm using a Synergy H1 microplate reader. Mitochondrial depolarization was expressed as the green/red fluorescence emission ratio and normalized relative to control values.

### 2.8. Statistical Analysis

All results are expressed as means ± standard deviation (SD). Normality of data distribution was assessed, and homogeneity of variances was evaluated using both Bartlett’s and Brown–Forsythe tests. Statistical significance among the groups was determined using one-way analysis of variance (ANOVA), followed by Tukey’s post hoc test for multiple comparisons. Statistical analyses were performed using GraphPad Prism version 7.04 for Windows (GraphPad Software, San Diego, CA, USA). A *p*-value < 0.05 was considered statistically significant, corresponding to a 95% confidence interval.

## 3. Results

### 3.1. Ketamine and Sodium Selenite Increased TRPV1-Associated Cytosolic Ca^2+^ Responses in SH-SY5Y Cells

**Figure 2:**
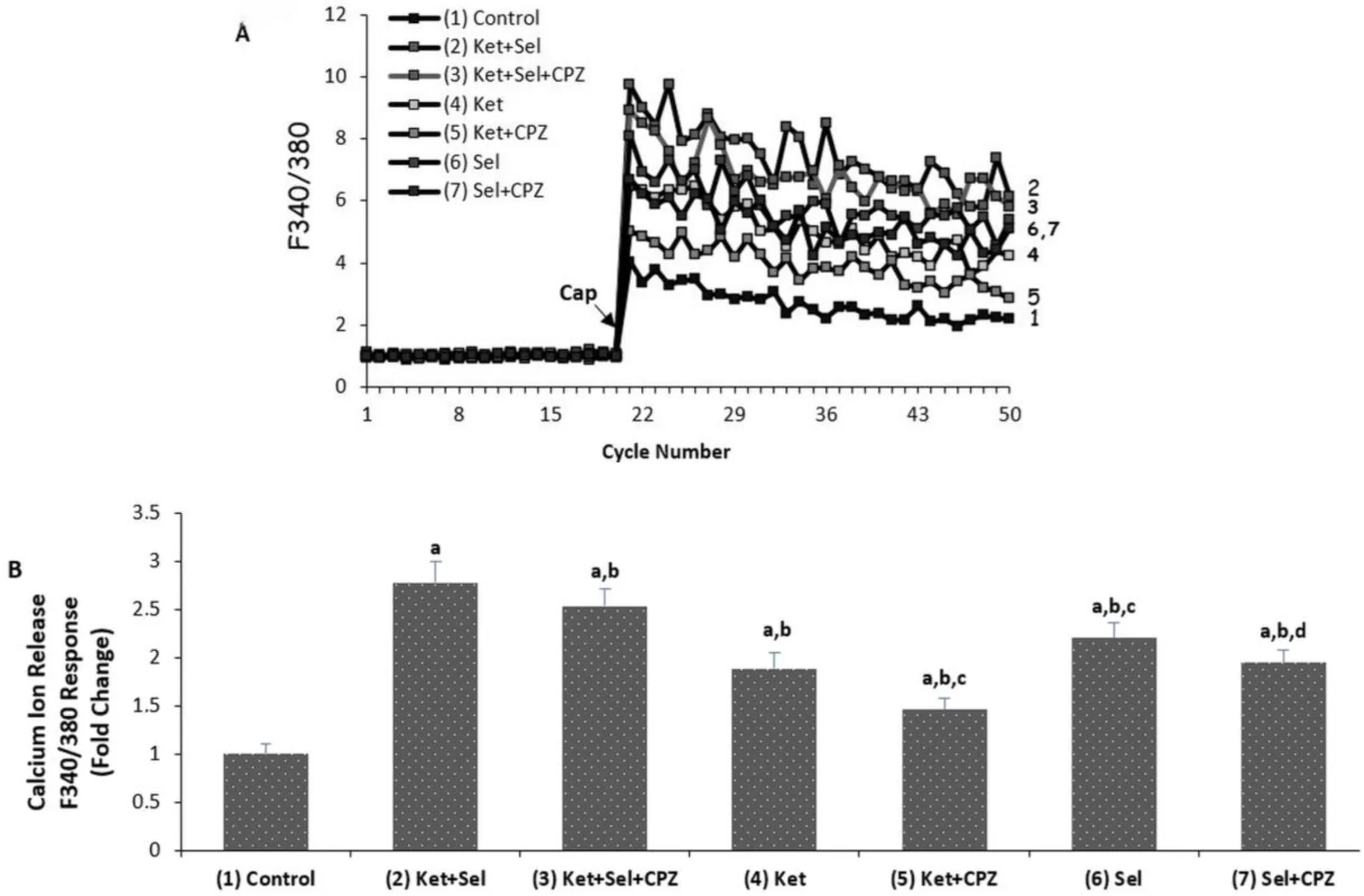
Effects of ketamine and sodium selenite on TRPV1-associated cytosolic Ca^2+^ responses in SH-SY5Y cells. (**A**) Representative F340/F380 traces after capsaicin stimulation at the 20th acquisition cycle. (**B**) Quantitative analysis of Ca^2+^ responses expressed as fold change. Cells were treated with ketamine (Ket, 10 µM, 48 h), sodium selenite (Sel, 500 nM, 48 h), and/or capsazepine (CPZ, 0.1 mM, 30 min). Data are presented as mean ± SD (*n* = 3). The experiments were performed using a total of 3 samples (*n* = 3) derived from three independent biological materials. a *p* < 0.001 vs. Control; b *p* < 0.001 vs. Ket+Sel; c *p* < 0.001 vs. Ket; d *p* < 0.001 vs. Sel.

The effects of ketamine and sodium selenite on cytosolic Ca^2+^ responses are shown in Figure 2A,B. Cap stimulation induced a marked increase in Fura-2 fluorescence ratio in treated SH-SY5Y cells. Cytosolic Ca^2+^ responses were significantly higher in the Ket+Sel, Ket, and Sel groups than in the control group (*p* < 0.001). The combined Ket+Sel group showed a greater Ca^2+^ response than either Ket or Sel alone (*p* < 0.001). Sel treatment produced a higher Ca^2+^ response than Ket treatment.

CPZ pretreatment attenuated cytosolic Ca^2+^ responses in the Ket+Sel+CPZ, Ket+CPZ, and Sel+CPZ groups compared with the corresponding groups without CPZ (*p* < 0.001), indicating that TRPV1 channel activity contributed to the observed intracellular Ca^2+^ response.

### 3.2. Ketamine and Sodium Selenite Increased Apoptosis and Intracellular ROS Production

**Figure 3:**
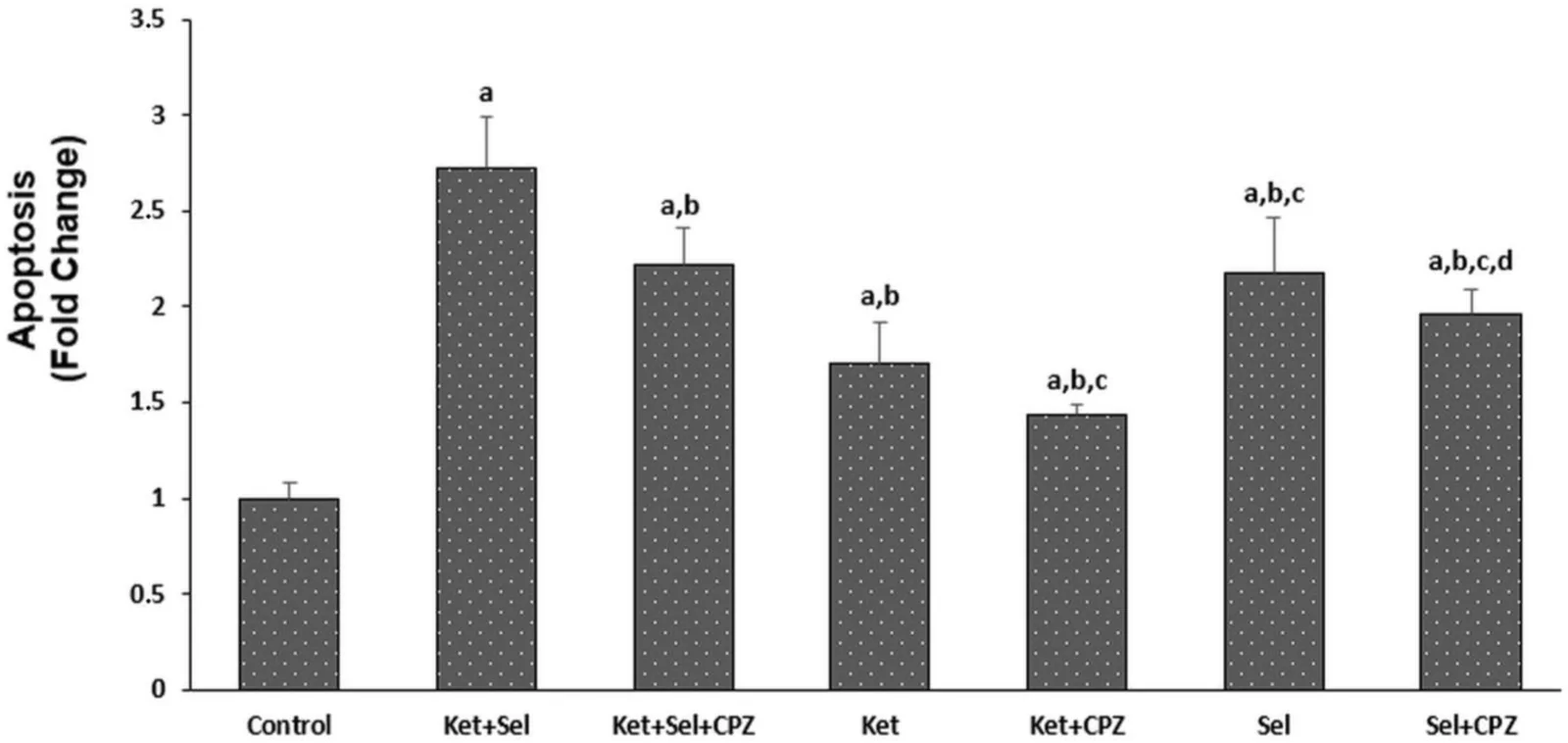
Effects of ketamine and sodium selenite on apoptosis in SH-SY5Y cells. Cells were treated with Ket (10 µM, 48 h), Sel (500 nM, 48 h), and/or CPZ (0.1 mM, 30 min) and stimulated with capsaicin (Cap, 0.1 mM, 10 min). Data are presented as mean ± SD. The experiments were performed using a total of 10 samples (*n* = 10) derived from three independent biological materials (3 + 3 + 4). a *p* < 0.001 vs. Control; b *p* < 0.001 vs. Ket+Sel; c *p* < 0.001 vs. Ket; d *p* < 0.001 vs. Sel.

**Figure 4:**
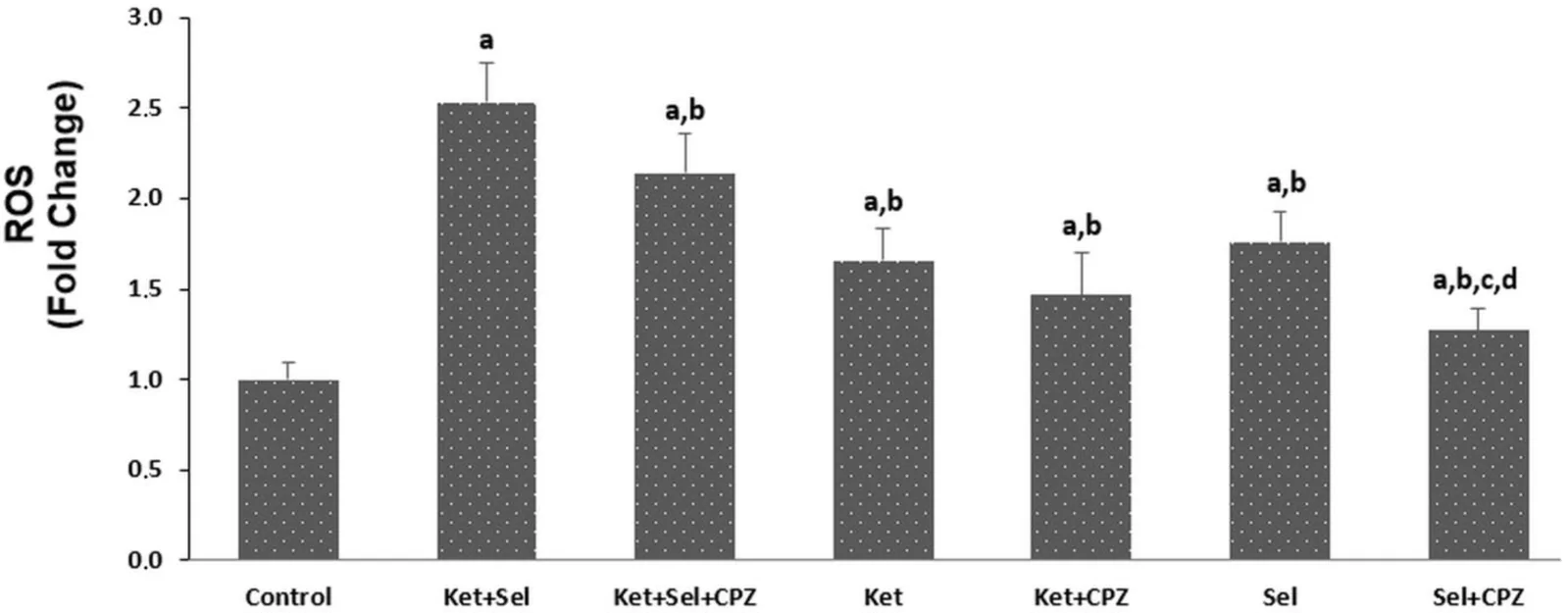
Effects of ketamine and sodium selenite on intracellular reactive oxygen species (ROS) production in SH-SY5Y cells. Cells were treated with Ket (10 µM, 48 h), Sel (500 nM, 48 h), and/or CPZ (0.1 mM, 30 min) and stimulated with Cap (0.1 mM, 10 min). Data are presented as mean ± SD. The experiments were performed using a total of 10 samples (*n* = 10) derived from three independent biological materials (3 + 3 + 4). a *p* < 0.001 vs. Control; b *p* < 0.001 vs. Ket+Sel; c *p* < 0.001 vs. Ket; d *p* < 0.001 vs. Sel.

Apoptosis and ROS production are presented in Figure 3 and Figure 4. Compared with the control group, Ket+Sel, Ket, and Sel treatments significantly increased apoptosis and intracellular ROS production in SH-SY5Y cells (*p* < 0.001). The combined Ket+Sel group produced higher apoptotic and ROS responses than the single-agent Ket and Sel groups (*p* < 0.001). Apoptosis levels were higher in the Sel group than in the Ket group, whereas ROS levels did not show a statistically significant difference between the Ket and Sel groups.

CPZ pretreatment reduced apoptosis and ROS responses in the corresponding CPZ-treated groups compared with their non-CPZ counterparts (*p* < 0.001), supporting a role for TRPV1-associated signaling in treatment-induced oxidative stress and cell death.

### 3.3. Ketamine and Sodium Selenite Increased Mitochondrial Depolarization and Caspase Activation

**Figure 5:**
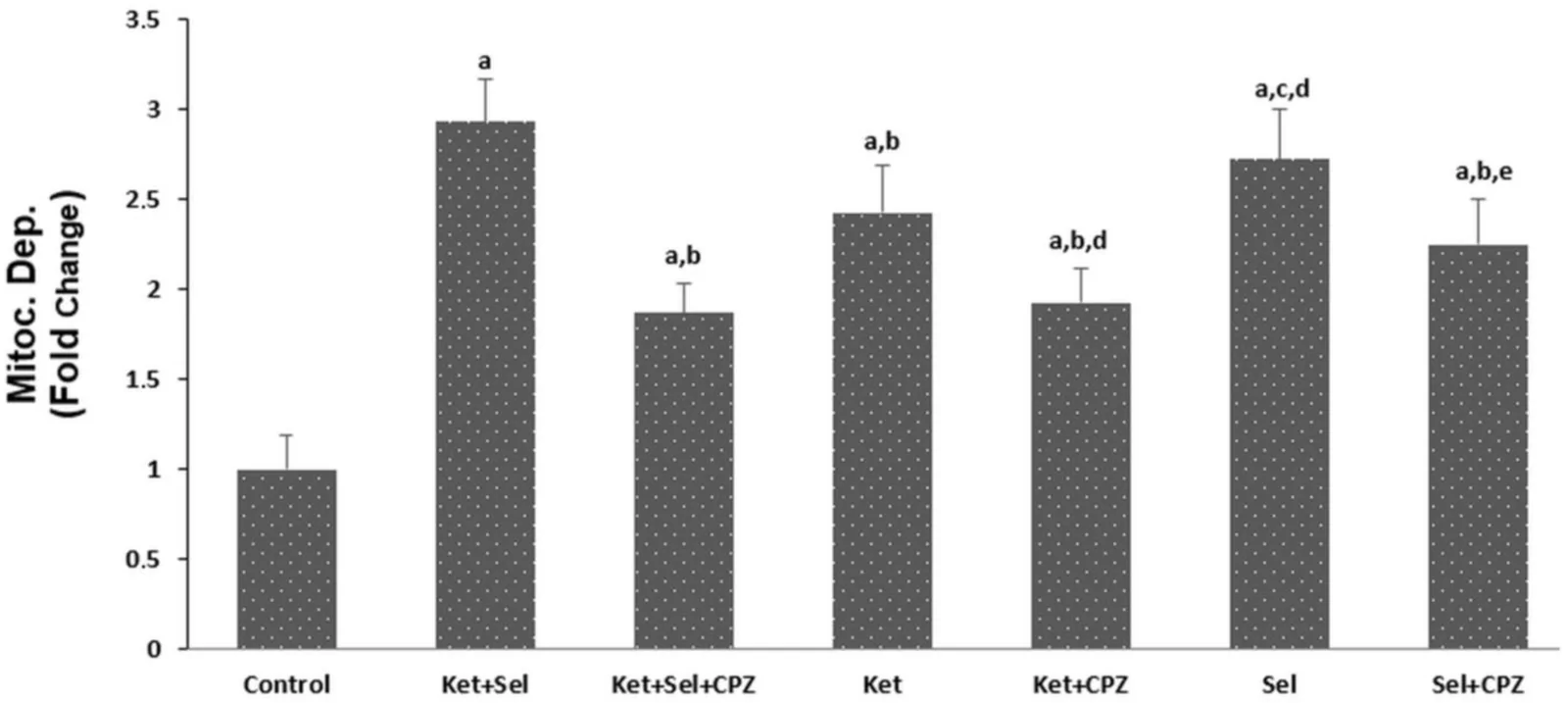
Effects of ketamine and sodium selenite on mitochondrial depolarization in SH-SY5Y cells. Cells were treated with Ket (10 µM, 48 h), Sel (500 nM, 48 h), and/or CPZ (0.1 mM, 30 min) and stimulated with Cap (0.1 mM, 10 min). Data are presented as mean ± SD. The experiments were performed using a total of 10 samples (*n* = 10) derived from three independent biological materials (3 + 3+ 4). a *p* < 0.001 vs. Control; b *p* < 0.001 and c *p* < 0.05 vs. Ket+Sel; d *p* < 0.001 vs. Ket; e *p* < 0.001 vs. Sel.

**Figure 6:**
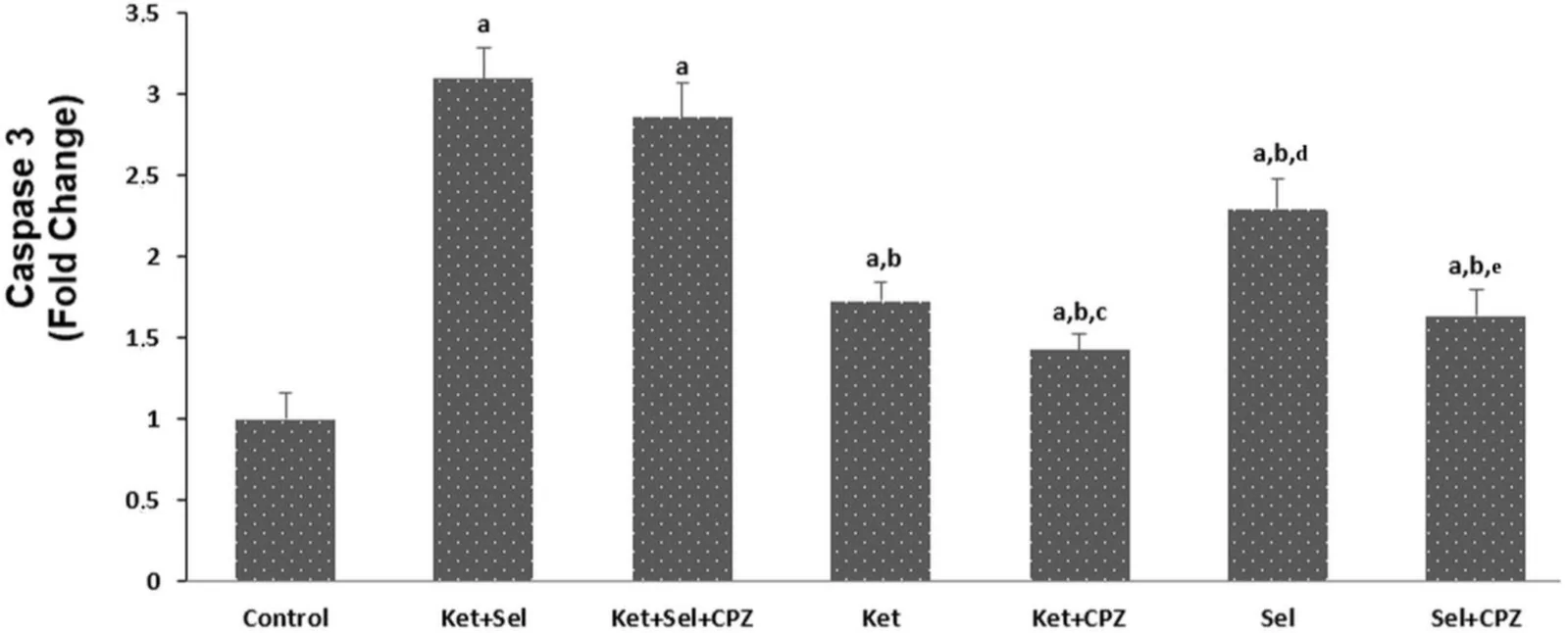
Effects of ketamine and sodium selenite on caspase-3 activity in SH-SY5Y cells. Cells were treated with Ket (10 µM, 48 h), Sel (500 nM, 48 h), and/or CPZ (0.1 mM, 30 min) and stimulated with Cap (0.1 mM, 10 min). Data are presented as mean ± SD. The experiments were performed using a total of 10 samples (*n* = 10) derived from three independent biological materials (3 + 3 + 4). a *p* < 0.001 vs. Control; b *p* < 0.001 vs. Ket+Sel; c *p* < 0.05 and d *p* < 0.001 vs. Ket; e *p* < 0.001 vs. Sel.

**Figure 7:**
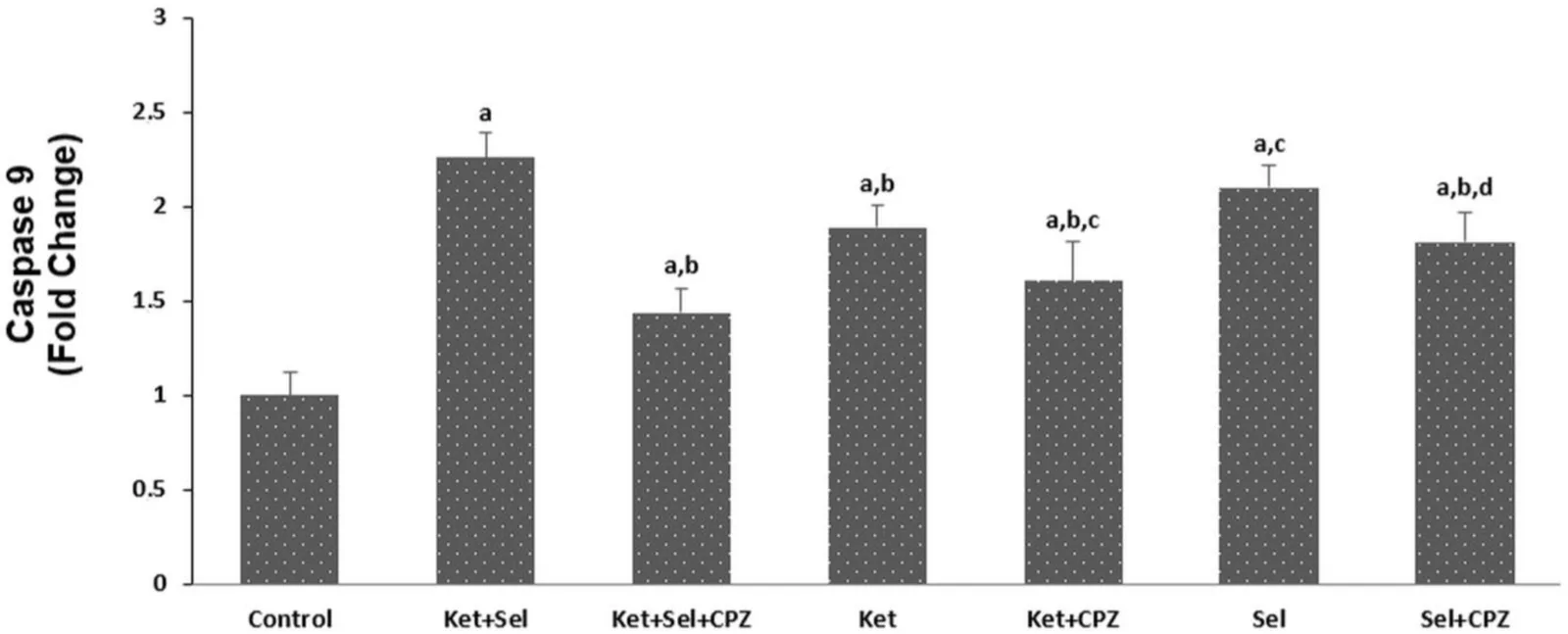
Effects of ketamine and sodium selenite on caspase-9 activity in SH-SY5Y cells. Cells were treated with Ket (10 µM, 48 h), Sel (500 nM, 48 h), and/or CPZ (0.1 mM, 30 min) and stimulated with Cap (0.1 mM, 10 min). Data are presented as mean ± SD. The experiments were performed using a total of 10 samples (*n* = 10) derived from three independent biological materials (3 + 3 + 4). a *p* < 0.001 vs. Control; b *p* < 0.001 vs. Ket+Sel; c *p* < 0.001 vs. Ket; d *p* < 0.001 vs. Sel.

Mitochondrial depolarization and caspase-3 and caspase-9 activities are shown in Figure 5, Figure 6 and Figure 7. Ket+Sel, Ket, and Sel groups showed significantly higher mitochondrial depolarization and caspase-3 and caspase-9 activities than the control group (*p* < 0.001). The Ket+Sel group generally showed the highest values, indicating enhanced mitochondrial dysfunction and caspase-dependent apoptotic signaling after combined treatment.

Compared with single-agent groups, the combined treatment increased mitochondrial depolarization and caspase activation. Sel treatment produced higher caspase-3, caspase-9, and mitochondrial depolarization responses than Ket alone. CPZ pretreatment reduced mitochondrial depolarization and caspase activities in the Ket+CPZ and Sel+CPZ groups compared with the corresponding non-CPZ groups. In the combined-treatment condition, CPZ resulted in a numerical reduction in caspase-3 activity; however, this difference did not reach statistical significance. These findings do not establish a definitive TRPV1-specific causal mechanism. Rather, they should be interpreted as being compatible with the involvement of TRPV1-associated signaling.

## 4. Discussion

The present study shows that ketamine and sodium selenite, administered alone and in combination, increased capsaicin-evoked intracellular Ca^2+^ responses, apoptosis, ROS production, mitochondrial membrane depolarization, and caspase-3 and caspase-9 activities relative to control values in SH-SY5Y neuroblastoma cells. The combined treatment produced greater responses than either agent alone for most endpoints. CPZ pretreatment attenuated the Ca^2+^ response and several downstream oxidative and apoptotic endpoints, supporting a contribution of CPZ-sensitive signaling consistent with TRPV1 involvement. However, these pharmacological findings do not establish that the observed effects were mediated exclusively by TRPV1.

TRP channels participate in cancer-related processes, including proliferation, apoptosis, metabolism, migration, and invasion [11]. Among these channels, TRPV1 is a Ca^2+^-permeable channel whose activation can engage oxidative and apoptotic signaling. Previous experimental studies have linked TRPV1-associated Ca^2+^ signaling to oxidative stress and caspase activation in a neuronal model [14] and to ROS-dependent cytotoxicity in gastric cancer cells [15]. Consistent with these observations, capsaicin-evoked Ca^2+^ responses increased following treatment with ketamine, sodium selenite, or their combination in SH-SY5Y cells, whereas CPZ pretreatment attenuated these responses. These findings are consistent with a contribution of TRPV1-associated Ca^2+^ signaling to the observed cellular effects. Phospholipase C (PLC)/IP_3_-dependent mobilization of Ca^2+^ from intracellular stores represents an additional mechanism contributing to intracellular Ca^2+^ signaling, alongside Ca^2+^ entry through plasma-membrane channels [12]. Altered PLC signaling has also been implicated in nervous-system and glioma-related cellular processes [12,13]. Because PLC-dependent signaling was not directly examined in the present study, the Ca^2+^ responses reported here should not be attributed to a single channel or signaling pathway.

Ketamine is best characterized as a non-competitive NMDA receptor antagonist, although it also interacts with voltage-gated ion channels and several neurotransmitter receptor systems [4,5]. Experimental evidence indicates that its cellular effects are strongly dependent on concentration, exposure duration, and cell type. In HepG2 cells, S-(+)-ketamine induced apoptosis through a Bax–mitochondria–caspase pathway [7], whereas in H4 neuroglioma and A549 lung cancer cells, ketamine increased ROS and cleaved caspase-3 expression and inhibited cell proliferation [8]. In SH-SY5Y cells, exposure to 1000 µM ketamine for 12 h reduced cell viability and induced apoptosis and endoplasmic reticulum stress [26], whereas exposure to 0.39–6.25 µM ketamine for 24 h produced relatively low toxicity and no significant changes in ROS, malondialdehyde, or glutathione levels [10]. In the present study, under capsaicin-stimulated assay conditions, 10 µM ketamine for 48 h increased intracellular Ca^2+^ responses, apoptosis, ROS production, mitochondrial membrane depolarization, and caspase-3 and caspase-9 activities relative to control values. These differences across studies likely reflect variations in concentration, exposure duration, cell type, and assay conditions. The partial attenuation of the present responses by CPZ further supports a contribution of CPZ-sensitive signaling to the ketamine-associated cellular effects.

Selenium compounds exert concentration- and context-dependent biological effects. Selenium is essential for redox regulation and antioxidant defense [16], whereas selenite may exert cytoprotective or pro-apoptotic effects depending on concentration and experimental context [17,18,20]. In the present study, sodium selenite (500 nM, 48 h), under capsaicin-stimulated assay conditions, increased intracellular Ca^2+^ responses, apoptosis, ROS production, mitochondrial membrane depolarization, and caspase-3 and caspase-9 activities in SH-SY5Y cells. By contrast, Wang et al. reported that selenite attenuated cadmium-induced ROS production, apoptosis, and cytotoxicity in the same cell line by increasing thioredoxin reductase 1 activity and expression [18]. The contrasting findings may reflect differences in the prevailing redox challenge, concentration, exposure duration, and assay conditions. In other models, selenium reduced TRPV1-associated Ca^2+^ entry and oxidative stress in neutrophils [19], whereas in MCF-7 cells it reduced capsaicin-evoked TRPV1-associated Ca^2+^ responses while increasing mitochondrial membrane depolarization, apoptosis, and caspase-3 and caspase-9 activities [20]. Collectively, these findings indicate that the cellular consequences of selenium exposure are context-dependent and do not follow a uniform antioxidant or pro-oxidant pattern.

The combined Ket+Sel treatment produced greater responses than either single agent for most endpoints. These findings suggest that ketamine and sodium selenite may engage partially overlapping Ca^2+^-dependent oxidative and mitochondrial apoptotic processes. However, because formal drug-interaction analysis was not performed, the interaction cannot be classified as additive or synergistic. Accordingly, the combined effect is best described as an enhanced response. The partial attenuation of several endpoints by CPZ indicates that a component of the response was CPZ-sensitive and is compatible with TRPV1 involvement; however, the pharmacological design does not establish target specificity. Residual responses after CPZ pretreatment further indicate that the observed effects were not fully CPZ-sensitive and may involve additional redox, mitochondrial, or ion-channel mechanisms.

Taken together, the findings support a working model in which capsaicin-evoked, CPZ-sensitive Ca^2+^ responses are associated with increased ROS production, mitochondrial membrane depolarization, and increased caspase-9 and caspase-3 activities. Because the temporal sequence and causal dependence of these events were not directly examined, the model should be regarded as a pharmacologically informed, hypothesis-generating framework rather than as evidence of a linear or exclusively TRPV1-mediated pathway.

This study has several limitations. First, the experiments were conducted in a single neuroblastoma cell line without validation in additional neuroblastoma models or comparison with non-malignant neuronal or neural crest-derived cells. Second, TRPV1 involvement was inferred from pharmacological modulation with capsaicin and CPZ rather than from direct molecular or electrophysiological evidence. Both capsaicin and CPZ were used at the relatively high concentration of 0.1 mM in the present study. At this concentration, capsaicin and capsazepine have been shown in other experimental systems to inhibit native and recombinant T-type voltage-gated Ca^2+^ channels [27]. Therefore, potential off-target effects cannot be excluded, and CPZ sensitivity should not be regarded as specific evidence of TRPV1 mediation. Future studies should incorporate orthogonal approaches, including genetic silencing or knockout, protein-level confirmation, and electrophysiological characterization of TRPV1. Third, only one concentration of each agent and a single 48 h ketamine/selenite exposure period were evaluated, precluding concentration–response and time-course analyses. Fourth, the Ca^2+^ experiments were based on three independent biological replicates; additional independent replication would improve the precision and robustness of the estimates. Fifth, formal drug-interaction analysis was not performed; therefore, the enhanced combined response cannot be classified as additive or synergistic. Finally, the absence of in vivo validation limits conclusions regarding the biological and translational relevance of the tested concentrations.

In conclusion, under the tested in vitro conditions, ketamine and sodium selenite, alone and particularly in combination, increased capsaicin-evoked intracellular Ca^2+^ responses and enhanced apoptosis, ROS production, mitochondrial membrane depolarization, and caspase-3 and caspase-9 activities in SH-SY5Y neuroblastoma cells. The attenuation of several endpoints by CPZ indicates a CPZ-sensitive component compatible with TRPV1 involvement; however, these pharmacological findings do not establish selective or exclusive TRPV1 mediation. These results support further mechanistic investigation using orthogonal validation of TRPV1 involvement, concentration–response and time-course designs, formal drug-interaction analysis, and additional neuroblastoma and non-malignant cellular models.

## Data Availability

The original contributions presented in this study are included in the article. Further inquiries can be directed to the corresponding author.

## References

[1] Matthay K.K., Maris J.M., Schleiermacher G., Nakagawara A., Mackall C.L., Diller L., Weiss W.A. (2016). Neuroblastoma. Nat. Rev. Dis. Primers.

[2] Pinto N.R., Applebaum M.A., Volchenboum S.L., Matthay K.K., London W.B., Ambros P.F., Nakagawara A., Berthold F., Schleiermacher G., Park J.R. (2015). Advances in Risk Classification and Treatment Strategies for Neuroblastoma. J. Clin. Oncol..

[3] Maris J.M., Hogarty M.D., Bagatell R., Cohn S.L. (2007). Neuroblastoma. Lancet.

[4] Zanos P., Moaddel R., Morris P.J., Riggs L.M., Highland J.N., Georgiou P., Pereira E.F.R., Albuquerque E.X., Thomas C.J., Zarate C.A. (2018). Ketamine and Ketamine Metabolite Pharmacology: Insights into Therapeutic Mechanisms. Pharmacol. Rev..

[5] Schnoebel R., Wolff M., Peters S.C., Bräu M.E., Scholz A., Hempelmann G., Olschewski H., Olschewski A. (2005). Ketamine Impairs Excitability in Superficial Dorsal Horn Neurones by Blocking Sodium and Voltage-Gated Potassium Currents. Br. J. Pharmacol..

[6] Ikonomidou C., Bosch F., Miksa M., Bittigau P., Vöckler J., Dikranian K., Tenkova T.I., Stefovska V., Turski L., Olney J.W. (1999). Blockade of NMDA Receptors and Apoptotic Neurodegeneration in the Developing Brain. Science.

[7] Lee S.-T., Wu T.-T., Yu P.-Y., Chen R.-M. (2009). Apoptotic Insults to Human HepG2 Cells Induced by S-(+)-Ketamine Occurs through Activation of a Bax-Mitochondria-Caspase Protease Pathway. Br. J. Anaesth..

[8] Saito J., Zao H., Wu L., Iwasaki M., Sun Q., Hu C., Ishikawa M., Hirota K., Ma D., ESA-IC Onco-Anaesthesiology Research Group (2023). “Anti-Cancer” Effect of Ketamine in Comparison with MK801 on Neuroglioma and Lung Cancer Cells. Eur. J. Pharmacol..

[9] Wu K.-C., Liao K.-S., Yeh L.-R., Wang Y.-K. (2022). Drug Repurposing: The Mechanisms and Signaling Pathways of Anti-Cancer Effects of Anesthetics. Biomedicines.

[10] Jurič A., Lovaković B.T., Zandona A., Rašić D., Češi M., Pizent A., Neuberg M., Canjuga I., Katalinić M., Vrdoljak A.L. (2023). The Effects of Ketamine on Viability, Primary DNA Damage, and Oxidative Stress Parameters in HepG2 and SH-SY5Y Cells. Arh. Hig. Rada Toksikol..

[11] Fels B., Bulk E., Pethő Z., Schwab A. (2018). The Role of TRP Channels in the Metastatic Cascade. Pharmaceuticals.

[12] Rusciano I., Marvi M.V., Owusu Obeng E., Mongiorgi S., Ramazzotti G., Follo M.Y., Zoli M., Morandi L., Asioli S., Fabbri V.P. (2021). Location-Dependent Role of Phospholipase C Signaling in the Brain: Physiology and Pathology. Adv. Biol. Regul..

[13] Ratti S., Marvi M.V., Mongiorgi S., Obeng E.O., Rusciano I., Ramazzotti G., Morandi L., Asioli S., Zoli M., Mazzatenta D. (2022). Impact of Phospholipase C Β1 in Glioblastoma: A Study on the Main Mechanisms of Tumor Aggressiveness. Cell Mol. Life Sci..

[14] Ozdamar Unal G., Demirdas A., Nazıroglu M., Ovey I.S. (2022). Agomelatine Attenuates Calcium Signaling and Apoptosis via the Inhibition of TRPV1 Channel in the Hippocampal Neurons of Rats with Chronic Mild Stress Depression Model. Behav. Brain Res..

[15] Liu L., Sun X., Guo Y., Ge K. (2022). Evodiamine Induces ROS-Dependent Cytotoxicity in Human Gastric Cancer Cells via TRPV1/Ca2+ Pathway. Chem. Biol. Interact..

[16] Zhang F., Li X., Wei Y. (2023). Selenium and Selenoproteins in Health. Biomolecules.

[17] Uğuz A.C., Nazıroğlu M., Espino J., Bejarano I., González D., Rodríguez A.B., Pariente J.A. (2009). Selenium Modulates Oxidative Stress-Induced Cell Apoptosis in Human Myeloid HL-60 Cells Through Regulation of Calcium Release and Caspase-3 and -9 Activities. J. Membrane Biol..

[18] Wang H., Sun S., Ren Y., Yang R., Guo J., Zong Y., Zhang Q., Zhao J., Zhang W., Xu W. (2023). Selenite Ameliorates Cadmium-Induced Cytotoxicity Through Downregulation of ROS Levels and Upregulation of Selenoprotein Thioredoxin Reductase 1 in SH-SY5Y Cells. Biol. Trace Elem. Res..

[19] Köse S.A., Nazıroğlu M. (2014). Selenium Reduces Oxidative Stress and Calcium Entry Through TRPV1 Channels in the Neutrophils of Patients with Polycystic Ovary Syndrome. Biol. Trace Elem. Res..

[20] Sakallı Çetin E., Nazıroğlu M., Çiğ B., Övey İ.S., Aslan Koşar P. (2017). Selenium Potentiates the Anticancer Effect of Cisplatin against Oxidative Stress and Calcium Ion Signaling-Induced Intracellular Toxicity in MCF-7 Breast Cancer Cells: Involvement of the TRPV1 Channel. J. Recept. Signal Transduct..

[21] Mak Y.T., Lam W.P., Lü L., Wong Y.W., Yew D.T. (2010). The Toxic Effect of Ketamine on SH-SY5Y Neuroblastoma Cell Line and Human Neuron. Microsc. Res. Tech..

[22] Freitas M., Alves V., Sarmento-Ribeiro A.B., Mota-Pinto A. (2011). Combined Effect of Sodium Selenite and Docetaxel on PC3 Metastatic Prostate Cancer Cell Line. Biochem. Biophys. Res. Commun..

[23] Martinez N.A., Ayala A.M., Martinez M., Martinez-Rivera F.J., Miranda J.D., Silva W.I. (2016). Caveolin-1 Regulates the P2Y2 Receptor Signaling in Human 1321N1 Astrocytoma Cells. J. Biol. Chem..

[24] Öz A., Çelik Ö. (2016). Curcumin Inhibits Oxidative Stress-Induced TRPM2 Channel Activation, Calcium Ion Entry and Apoptosis Values in SH-SY5Y Neuroblastoma Cells: Involvement of Transfection Procedure. Mol. Membr. Biol..

[25] Uguz A.C., Cig B., Espino J., Bejarano I., Naziroglu M., Rodríguez A.B., Pariente J.A. (2012). Melatonin Potentiates Chemotherapy-Induced Cytotoxicity and Apoptosis in Rat Pancreatic Tumor Cells. J. Pineal Res..

[26] Zhang R., Wang X., Xie Z., Cao T., Jiang S., Huang L. (2023). Lipoxin A4 Methyl Ester Attenuated Ketamine-Induced Neurotoxicity in SH-SY5Y Cells via Regulating Leptin Pathway. Toxicol. Vitr..

[27] McArthur J.R., Finol-Urdaneta R.K., Adams D.J. (2019). Analgesic Transient Receptor Potential Vanilloid-1-Active Compounds Inhibit Native and Recombinant T-Type Calcium Channels. Br. J. Pharmacol..

